# Profiles of prognostic alternative splicing signature in hepatocellular carcinoma

**DOI:** 10.1002/cam4.2875

**Published:** 2020-01-24

**Authors:** Fangming Wu, Qifeng Chen, Chaojun Liu, Xiaoran Duan, Jinlong Hu, Jian Liu, Huicun Cao, Wang Li, Hui Li

**Affiliations:** ^1^ Department of Comprehensive Intervention Henan Provincial People's Hospital Zhengzhou China; ^2^ Department of Comprehensive Intervention Zhengzhou University People's Hospital Zhengzhou China; ^3^ Department of Medical Imaging and Interventional Radiology Sun Yat‐sen University Cancer Center Guangzhou China; ^4^ Biotherapy Center the First Affiliated Hospital of Zhengzhou University Zhengzhou China; ^5^ Department of Oncology Henan Provincial People's Hospital Zhengzhou China

**Keywords:** alternative splicing, hepatocellular carcinoma, prognosis, TCGA, survival

## Abstract

Previous studies have demonstrated the role of abnormal alternative splicing (AS) in tumor progression. This study examines the prognostic index (PI) of alternative splices (ASs) in patients with hepatocellular carcinoma (HCC). The clinical features and splicing events of patients with HCC were downloaded from The Cancer Genome Atlas (TCGA). Differentially expressed AS (DEAS) were compared between HCC and adjacent normal samples. Univariate Cox regression analysis was used to determine changes in DEAS associated with overall survival (OS). A PI was generated from OS‐associated DEASs using Kaplan‐Meier curves, receiver operating characteristic (ROC) curves, multivariate Cox regression, and cluster analysis. Then, the correlation between DEASs and splicing factors was assessed, followed by functional and pathway enrichment analysis. We identified 34 163 ASs of 8985 genes in HCC, and 153 OS‐ASs were identified using univariate Cox regression analysis. Low‐ and high‐PI groups were determined based on the median “PI‐ALL” value according to significantly different survival (*P* = 2.2e − 16). The ROC curve of all PI (PI‐ALL) had an area under the curve (AUC) of 0.993 for survival status in patients with HCC. A potential regulatory network associated with prognosis of patients with HCC was established. Enrichment analysis also resulted in the identification of several pathways potentially associated with carcinogenesis and progression of HCC. Four clusters were identified that were associated with clinical features and prognosis. Our study generated comprehensive profiles of ASs in HCC. The interaction network and functional connections were used to elucidate the underlying mechanisms of AS in HCC.

## Graphical abstract

This study examines the prognostic index (PI) of alternative splices (ASs) in patients with hepatocellular carcinoma. The clinical features and splicing events of patients with HCC were downloaded from The Cancer Genome Atlas.

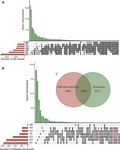

## 1 INTRODUCTION

Alternative splicing (AS), a critical posttranscriptional regulatory mechanism, modulates translation of mRNA isoforms and induces protein diversity, resulting in expansion of genome coding ability.^1^ During AS, a single RNA precursor could be spliced in diverse patterns to generate mRNA and protein variants with unique structures and functions.^2^ Alternative splicing has been detected in more than 95% of human genes, resulting in encoding of splicing variants under normal physiological conditions.^3^ However, tumor cells are capable of producing abnormal proteins with altered, missing, or inserted domains, resulting in carcinogenesis.^4^ Recent studies have confirmed the close relationship between abnormal AS and oncogenic processes, including therapeutic resistance, tumor progression, and metastasis.^4^, ^5^, ^6^, ^7^ Moreover, altered expression or mutations of splicing factors can result in global alterations in AS behavior, leading to oncogene and cancer pathway activation, and production of other cancer‐promoting splicing isoforms.^8^, ^9^, ^10^, ^11^ These findings suggest that tumor‐specific splice variants might be effective diagnostic, predictive, and prognostic biomarkers, and may also be promising therapeutic targets.

Hepatocellular carcinoma (HCC) has the fifth highest global mortality rate among all types of malignancies and this rate is rising rapidly.^12^ Transcript architecture regulated by AS in HCC was ignored until three recent analyses that report the entire profile of abnormal alternative splices (ASs) that participate in HCC tumorigenesis and progression.^13^, ^14^, ^15^ However, Zhu et al report alternative mRNA splicing in 377 cases of HCC, and incomplete information for patients followed up <30 days (one had no survival information and five were not followed up) might have resulted in false relationships in survival analysis.^13^ Moreover, cluster analysis was not performed in the studies by Zhu et al (2019a) or Wu et al (2019a). In addition, the studies by Wu et al (2019a) and Chen et al (2019c) did not find any differences in AS between HCC and non‐tumor samples. As such, it is important to further identify novel AS predictors for prognosis of patients with HCC.

Recent advances in deep sequencing techniques have allowed for evaluation of the role of splicing events in cancer biology. Using RNA‐seq data from TCGA, Ryan et al (16 constructed a TCGA SpliceSeq dataset (http://bioinformatics.mdanderson.org/TCGASpliceSeq/index.jsp), which provided access to data regarding splicing events in cancer. The SpliceSeq dataset consists of seven AS patterns: alternate terminator (AT), retained intron (RI), alternate acceptor site (AA), alternate promoter (AP), exon skip (ES), alternate donor site (AD), and mutually exclusive exons (ME).^17^, ^18^ The percent‐spliced‐in (PSI) value (range: 0‐1) was used for assessment of the transcript ratio between genes of interest and the seven AS patterns.

In this study, the roles of differential AS patterns were examined in patients with HCC using RNA‐seq data from TCGA with the goal of characterizing the prognostic value of HCC‐specific DEASs. In addition, a potential regulatory network was constructed to characterize the associations between splicing factors and ASs. The results of this study may contribute to understanding of the mechanisms underlying onset and progression of HCC.

## 2 MATERIALS AND METHODS

### 2.1 Data mining process and determination of DEASs

RNA sequencing data for the HCC cohort were obtained from TCGA data portal (https://portal.gdc.cancer.gov/). Alternative splicing data from patients with HCC were downloaded from TCGA SpliceSeq database to construct AS profiles for each patient by evaluating mRNA splicing patterns. To determined differences in ASs between HCC and adjacent samples, we identified significant DEASs and differentially expressed mRNAs between tumor and adjacent tissues with adjusted *P*‐value < .05 and log(fold change) >3/2 or <2/3. Differentially expressed AS were matched with differential expressed mRNAs.

### 2.2 Survival analysis

Patients with less than 30 days since last follow‐up were excluded were excluded.^15^ Three hundred forty‐three patients were enrolled based on the cutoff value (median value) of each AS. Univariate Cox regression analysis was performed to identify overall survival‐associated ASs (OS‐ASs). Multivariate Cox regression analysis was used to determine the independent prognostic value of the identified ASs, which contributed to establishment of predictive models. Prognostic indices (PI) were constructed via linear combination of expression level and the regression model (*β*). Kaplan‐Meier (KM) curves were generated to verify the abilities of the predictive models to differentiate between patients with prolonged OS and those whose OS was less. Receiver operator characteristic (ROC) curves were generated using the R project Consensus Cluster Plus package to estimate the time‐dependent ROC curve using censored data as a measure of predictive model efficiency.^19^ The area under the curve (AUC) of the ROC curve was calculated for each model. All patients underwent internal validation using bootstrap techniques. HCC patients were randomly assigned to three validation cohorts (75%, 80%, and 85% of the whole cohort). The mean AUCs for the three validation cohorts (AUC‐75%, AUC‐80%, and AUC‐85%) were calculated.

### 2.3 Construction of a correlation network

Data for 67 splicing factors were obtained from the “SpliceAid‐F” database (http://www.introni.it/splicing.html).^20^ Univariate Cox regression analysis was used to determine significant splicing factors. Kaplan‐Meier curve analysis was used to evaluate splicing factor‐related OS in patients with HCC. Spearman correlation tests were used to evaluate the relationships between the expression of splicing factor genes and PSI values of survival‐associated ASs. Correlation plots were generated using Cytoscape (3.6.0). Hub genes were subjected to functional enrichment analysis using cluster Profiler. Gene Ontology (GO) terms, Kyoto Encyclopedia of Genes and Genomes (KEGG) and Reactome pathways were enriched using ClueGo.

### 2.4 Cluster analysis

Patients were clustered into groups using the R package. Patients were further clustered into diverse groups based on consensus expression, followed by survival comparison among these groups. Chi‐squared tests were used to compare the distributions of survival, T stage, N stage, and M stage, and WHO grade status between the groups. Two‐sided *P*‐values < .05 were considered statistically significant.

## 3 RESULTS

### 3.1 Comprehensive analysis of ASs in the HCC cohort

**Figure 1:**
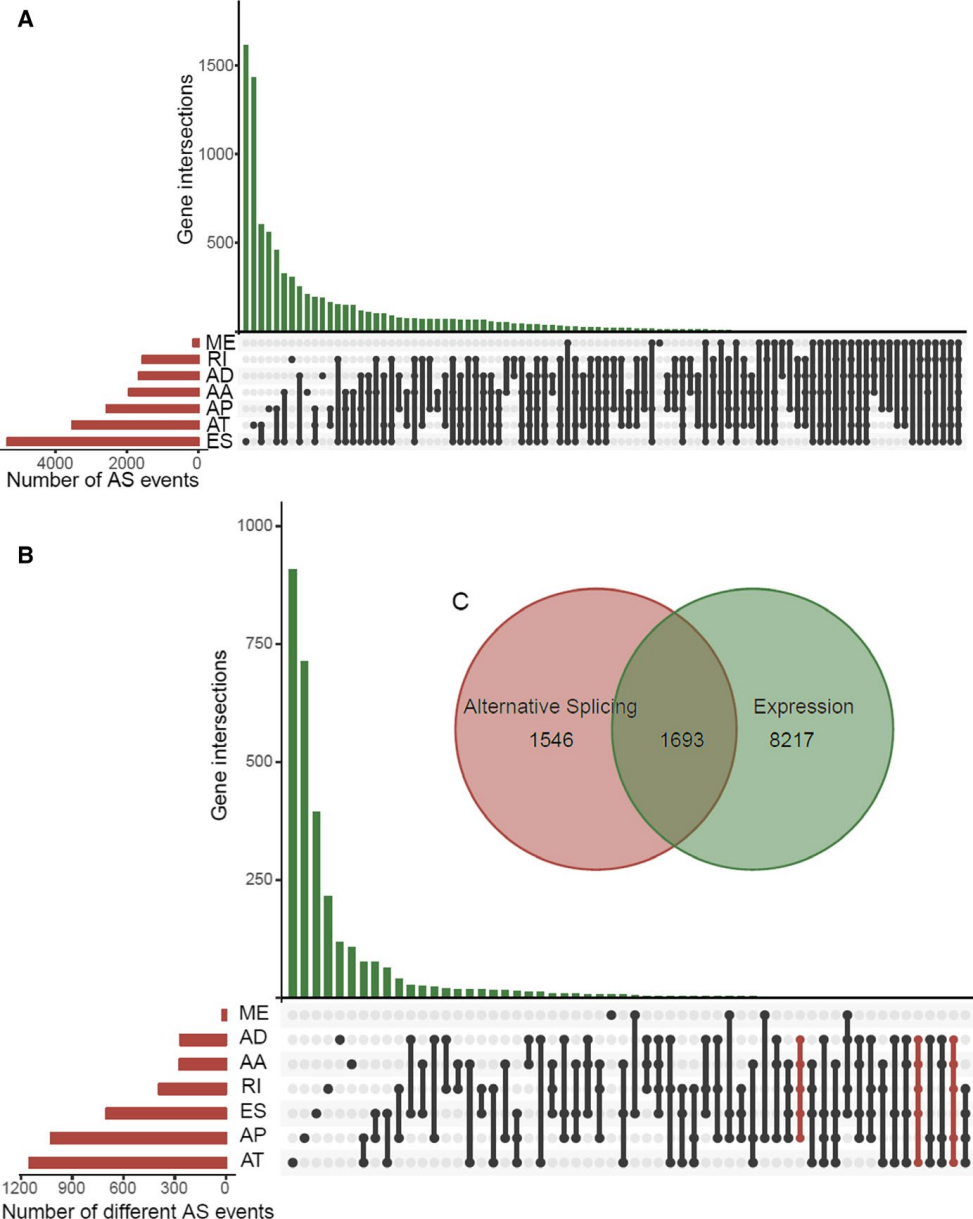
Overview of the seven types of alternative splicing in this study. UpSet plot of interactions of the seven types of alternative splicing (AS) events (A) with differential AS events in hepatocellular carcinoma (B). One single gene could have as many as four types of survival‐related AS. (C) Venn diagram showing that 1693 differential expressed ASs matched differentially expressed mRNA

In total, 34 163 mRNA splicing events were identified in 8985 genes. These events included 2666 AAs in 1937 genes, 2331 ADs in 1663 genes, 6352 APs in 2566 genes, 8087 ATs in 3532 genes, 12 327 ESs in 5343 genes, 137 MEs in 135 genes, and 2263 RIs in 1561 genes (Figure 1A). These results suggest a single gene could have up to five types of mRNA ASs. Of note, ES accounted for more than one‐third of all AS events.

As shown in Figure 1B, 4666 DEASs were detected in 3064 genes between HCC and normal samples. These included 4027 upregulated ASs and 639 downregulated ASs. Moreover, 9595 mRNAs were differentially expressed between HCC and normal samples, including 7005 upregulated genes and 2590 downregulated genes. A Venn diagram was constructed to depict overlap of DEASs and differentially expressed mRNAs (Figure 1C).

### 3.2 Survival‐related as events in the HCC cohort from TCGA

**Figure 2:**
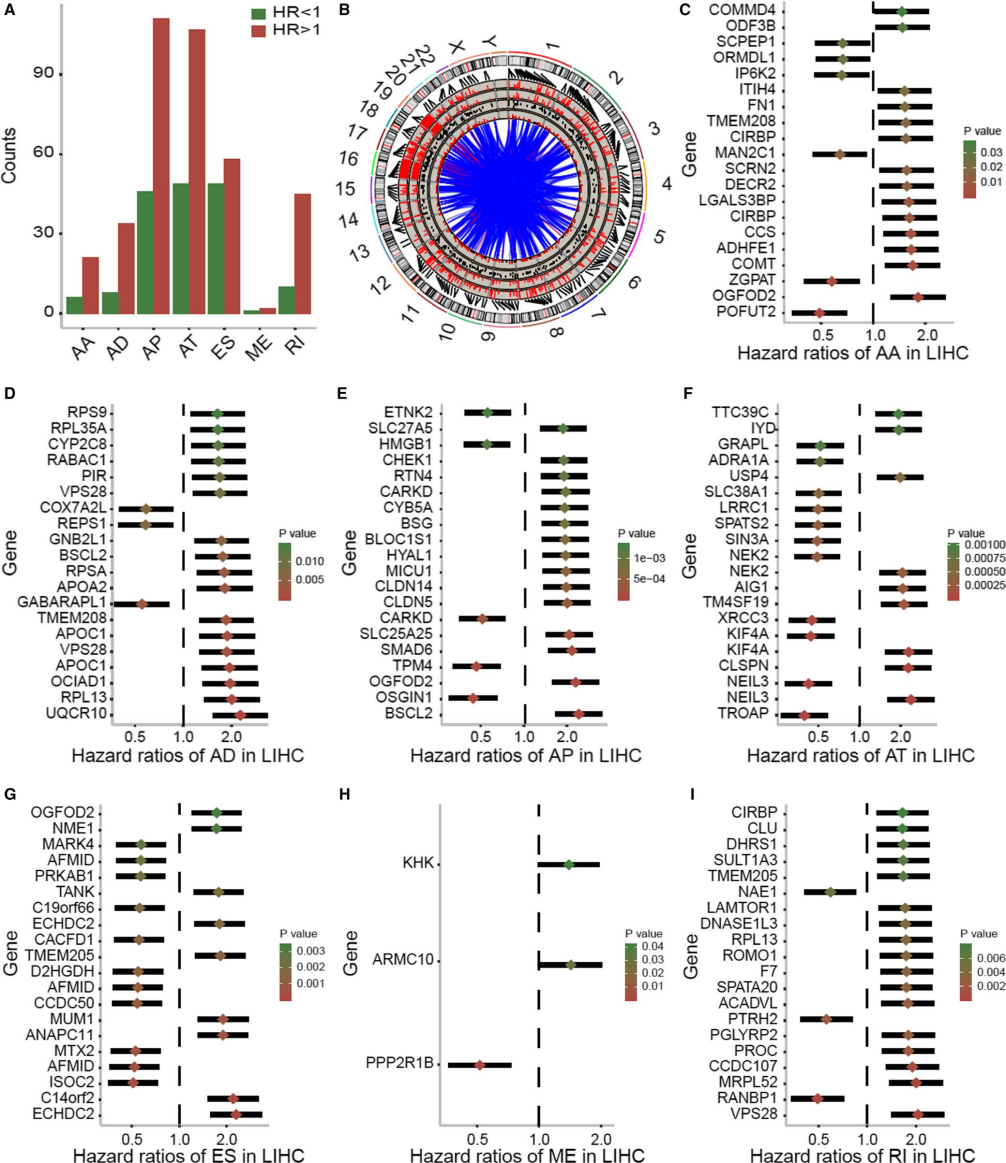
Overview of the seven types of survival‐related alternative splicing (AS). A, The number of prognosis‐related ASs. Green columns show favorable ASs; Red columns show the unfavorable ASs. B, Circos plots show the prognostic AS‐ related genes. The outer layer represents the chromosome model, and the inner layer illustrates the survival‐related AS genes. Red color represents genes are up‐regulated. Green color represents genes are down‐regulated. C‐I, Forrest plots of hazard ratios of survival‐related ASs in hepatocellular carcinoma

We examined the relationship between ASs and OS by performing univariate survival analyses Five hundred forty‐seven survival‐related ASs were identified in HCC (*P* < .05) with the following distribution:27 AA, 4 AD, 157 AP, 156 AT, 107 ES, 3 ME, and 55 RI. As shown in Figure 2A, most of the ASs were risk factors for reduced survival (hazard ratio [HR] > 1). Survival related alternatively spliced genes are summarized in Figure 2B. The top 20 survival‐related genes with the highest statistical significance among the seven types of AS are shown in Figure 2C‐I.

### 3.3 Prognostic predictors for HCC patients

The survival‐related ASs with the highest levels of significance among the seven types of AS were chosen as candidates. Multivariate Cox regression and establishment of a prognostic model were used to evaluate each of the candidate ASs, to eliminate any possible independent indicators in the prognostic gene model. The eight prognostic indices were abbreviated as follows: PI‐AA, PI‐AD, PI‐AP, PI‐AT, PI‐ES, PI‐ME, PI‐RI, and PI‐ALL.

**Figure 3:**
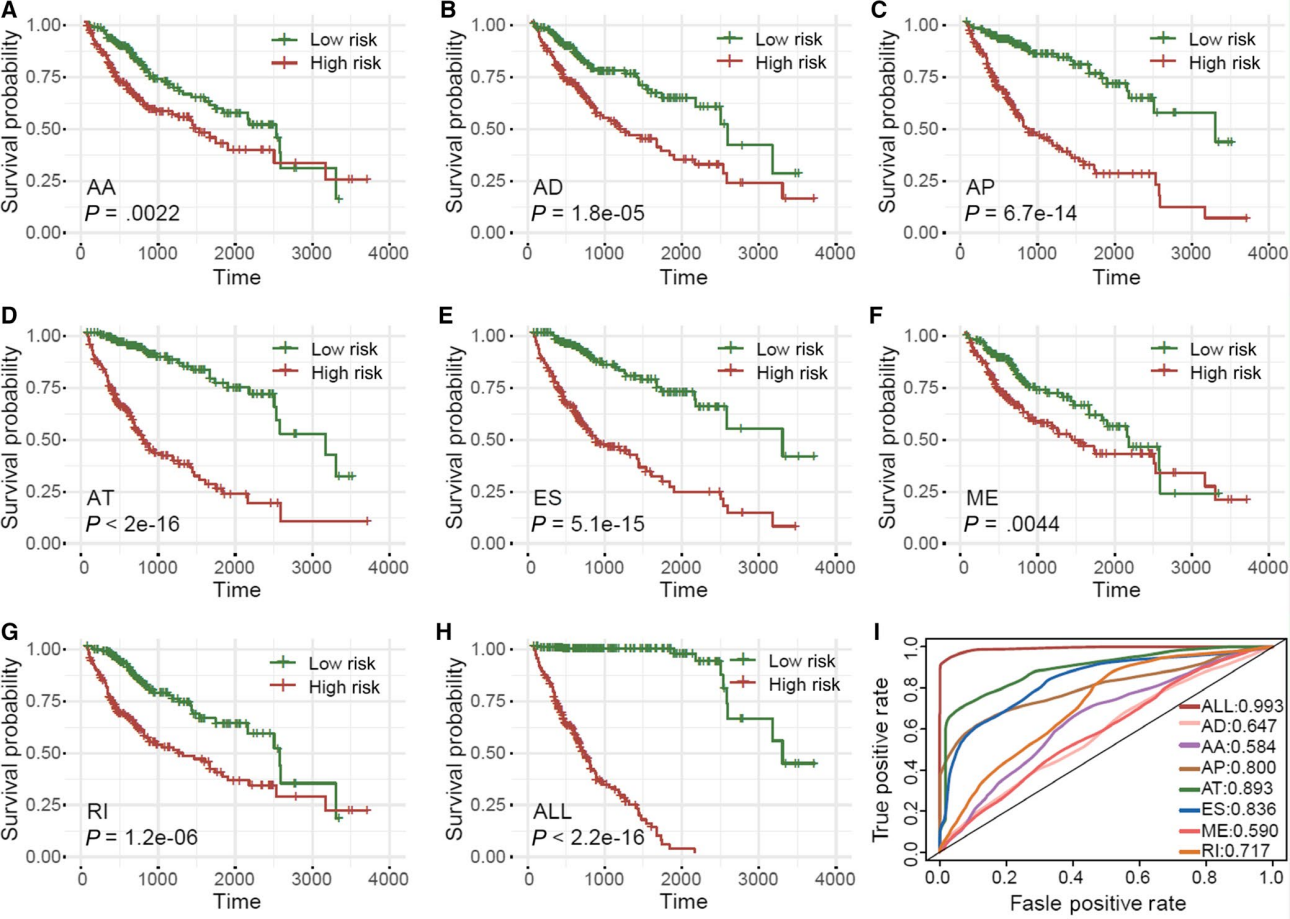
Survival analysis of the eight prognostic index (PI) types. A‐H, Kaplan‐Meier curves of PI to assess OS of patients with HCC following division into low‐ and high‐PI groups based on median PI value. I, Time‐dependent ROC curve of PI to predict survival status

As shown in Figure 3A‐H, patients with HCC were assigned to low‐ and high‐PI groups based on the median values of the eight PIs, and a KM curve analysis was performed. The low and high groups based on the median value of PI‐AT had the greatest statistical significance for association with OS (*P* = 2e−16), followed by PI‐ALL (*P* = 2.2e − 16), PI‐ES (*P* = 5.1e − 15), PI‐AP (*P* = 6.7e − 14), PI‐RI (*P* = 1.2e − 6), PI‐AD (*P* = 1.8e − 5), PI‐AA (*P* = .0022), and PI‐ME (*P* = .0044). Receiver operating characteristic curves verified that the AS‐ALL was the best prognostic predictor (AUC = 0.993)(Figure 3I). The mean AUCs were 0.986, 0.986, and 0.985 for AUC‐75%, AUC‐80%, and AUC‐85% cohorts respectively (Table S1).

### 3.4 Network of survival‐associated ASS

**Figure 4:**
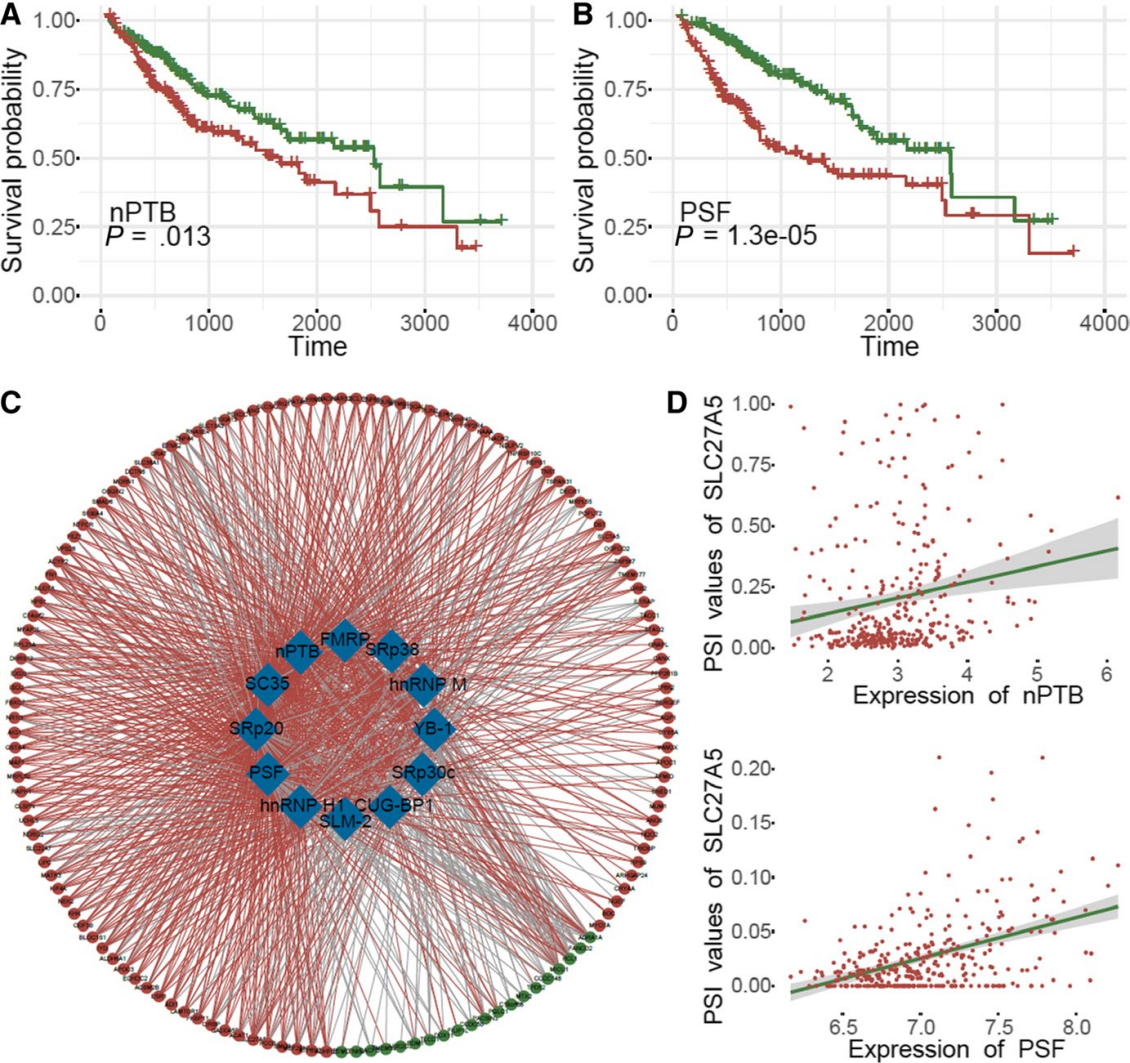
Correlation analysis between splicing factors and splicing events. A‐B, Kaplan‐Meier curves showed the correlations between the expression of the splicing factors, PSF and nPTB, with OS. C, Network of splicing factors and splicing events. Blue nodes indicated splicing factors, green nodes represent down‐regulated OS‐associated splicing events, red nodes indicate up‐regulated OS‐associated splicing events, red lines indicate positive correlations, and gray lines indicate negative correlations. D, Dot plot of correlations showing the association between the expression of nPTB and SLC27A5 PSI values, and the association between the expression of PSF and SLC27A5 PSI value

Using gene expression levels obtained from TCGA HCC RNA‐seq dataset, univariate Cox analysis showed that 12 out of 67 splicing factors were significantly associated with OS in HCC. Kaplan‐Meier curves of median values of the 12 splicing factors were generated to assess OS of patients with HCC (Figure 4A and Figure S1). Ten splicing factors were negatively associated with survival, including PSF and nPTB (Figure 4A,B). Moreover, the Spearman correlation was used to determine possible associations of survival‐related splicing factors with gene expression levels and PSI values. Significant correlations are summarized in the network depicted in Figure 4C. The expression of 12 survival‐related splicing factors (blue nodes) were significantly associated with 147 survival associated ASs in the correlation network of HCC, among which 20 were significantly downregulated ASs (green nodes) and 118 were significantly upregulated ASs (red nodes). The majority of prognostic ASs were positively correlated (red lines) with the expression of splicing factors (71.6%). Gray lines indicate ASs that were negatively correlated with splicing factor expression. The splicing factors PSF and nPTB were most frequently associated with ASs, accounting for 117 and 99 significant associations, respectively. AIG1, ATP6V0B, BLOC1S1, IYD, NDUFB2, RPL23A, and UCHL5 were significantly associated with 11 splicing factors, and SLC27A5 was associated with up to 12 splicing factors. The representative correlations are summarized in Figure 4D.

**Figure 5:**
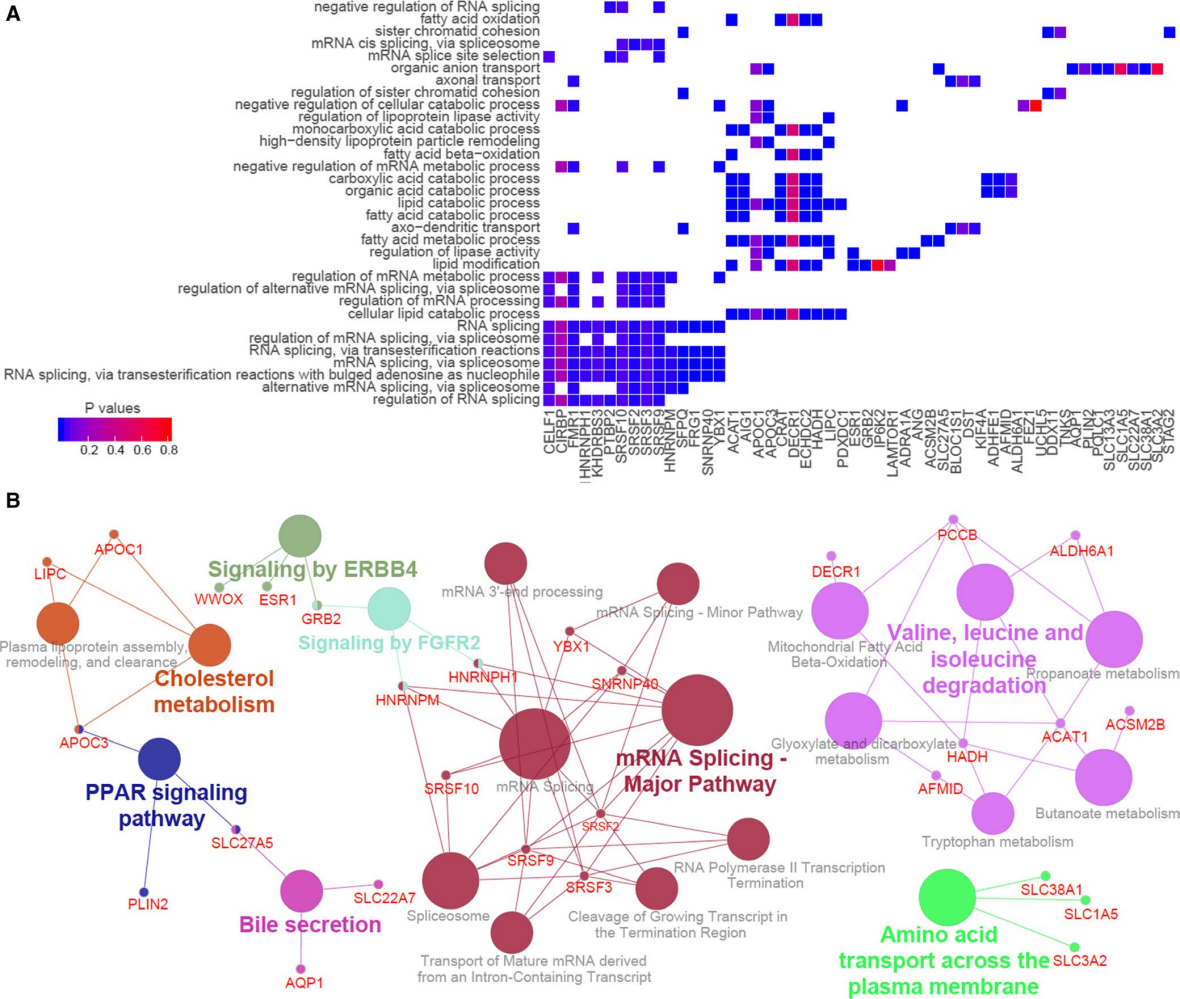
Enrichment analyses of genes associated with OS‐related splicing events. Gene ontology (A) and pathway analysis (B)

Functional enrichment analysis was performed to characterize the biological correlates of the constructed network. Gene ontology analysis revealed that the ASs and splicing factors identified in this study were enriched in fatty acid metabolic process, organic anion transport, cellular lipid catabolic process, and AS process (Figure 5A). Pathway analysis using KEGG and Reactome showed significant enrichment in pathways such as mRNA Splicing‐Major Pathway, PPAR signaling pathway, Signaling by FGFR2 (Figure 5B).

### 3.5 Cluster construction

**Figure 6:**
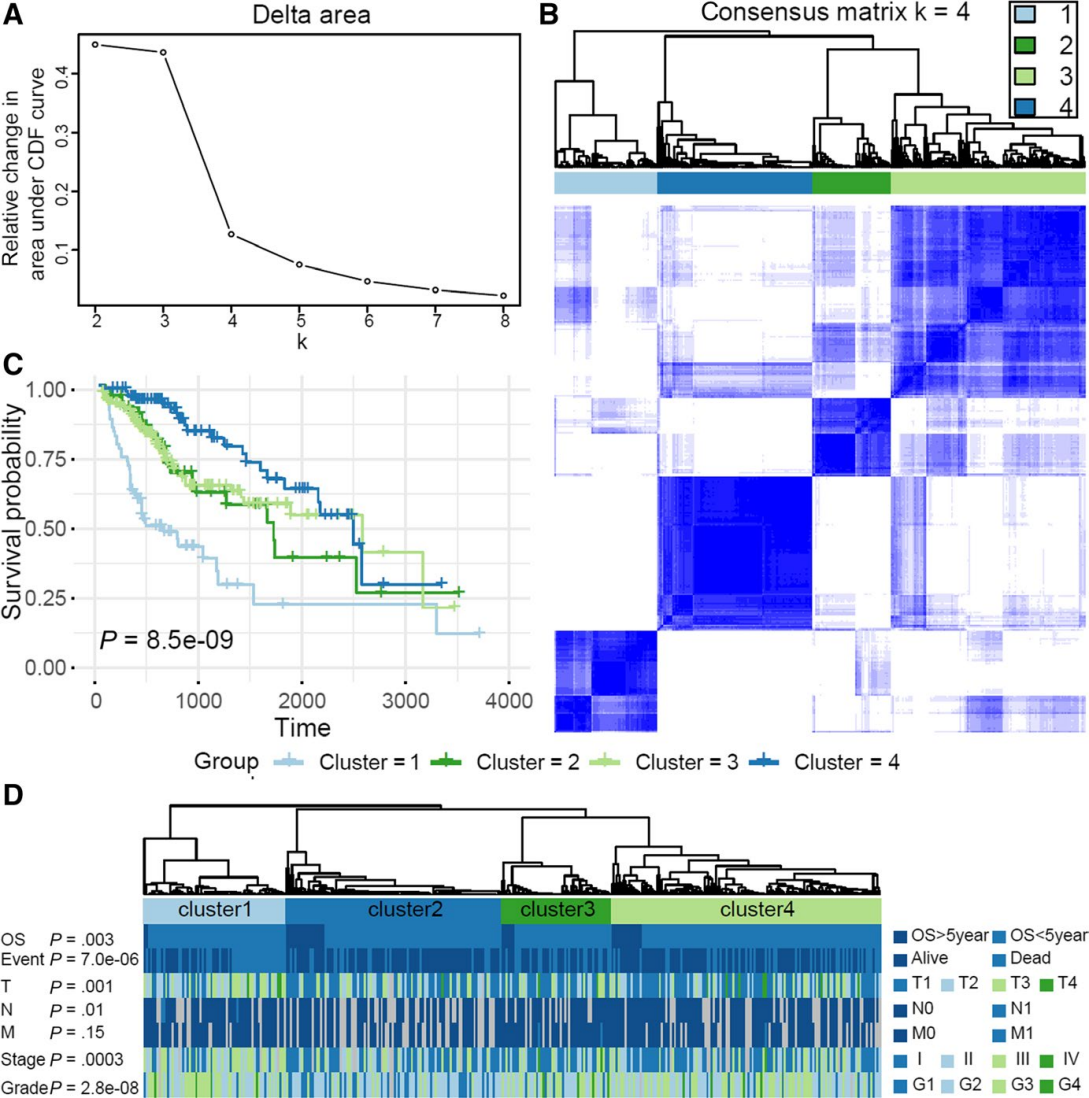
Identification of four clusters of patients with hepatocellular carcinoma (HCC) with distinct clinical features and outcomes using consensus clustering. A, Relative change in the area under the CDF curve for *k* = 2 to 8. B, Consensus clustering matrix for *k* = 4. C, Kaplan‐Meier curves for patients with HCC stratified by cluster. D, Heat map of the consensus matrix showing distinct clinical features and outcomes

Consensus clustering of AS events resulted in successful placement of HCC into four clusters with distinct AS characteristics and clinical outcomes. Based on the consensus results (Figure 6A‐B), *k* = 4 was considered an adequate selection. Therefore, patients were clustered into four subgroups, among which AS features and survival were compared. The subgroups were as follows: Cluster 1 (n = 66, 19.3%), Cluster 2 (n = 51, 14.9%), Cluster 3 (n = 125, 36.6%), and Cluster 4 (n = 100, 29.2%). Patients in the Cluster 1 subgroup had significantly worse prognoses (Figure 6C). The distributions of survival, T stage, N stage, and WHO grade status among the four clusters were significantly different (Figure 6D).

## 4 DISCUSSION

Aberrant pre‐mRNA AS has been previously identified as a contributor to tumor progression.^21^, ^22^ Recent advances in genome sequencing technologies and bioinformatics have resulted in massive publicly accessible databases that contain transcriptomic data for multiple tumor types. Among these databases, TCGA is considered the most comprehensive dataset for integrative genomic analysis of tumors, thereby contributing to the rapidly increasing in‐depth understanding of the molecular mechanisms of cancer.^23^, ^24^ In our current study, we mined data from the TCGA SpliceSeq database, and developed a univariate Cox regression model to identify survival‐associated ASs in HCC. We then determined PIs based on AS types, and established risk scores for prediction of prognoses of patients with HCC. Finally, we constructed a potential regulatory network of ASs and splicing factors, which identified multiple HCC‐specific and survival‐associated ASs. These ASs may be promising therapeutic targets for treatment of HCC.

Several studies have evaluated the role of AS in HCC. However, few studies have comprehensively assessed splicing events in HCC. Our study provided analysis of the prognostic potential of ASs in HCC that could be considered more extensive for the following reasons. First, we only selected patients for whom useful survival information was available. Second, we compared HCC tissue to adjacent samples to identify DEASs, which provided additional statistical power. Finally, we performed cluster analysis to characterize the role of AS in HCC.

Previous genome‐wide studies that evaluated the role of AS in HCC primarily focused on identification of “cancer‐specific” AS events via comparison between tumor tissues and normal controls. A recent study showed that the majority of alternative isoforms and tumor‐specific isoforms originated from abnormal splicing during hepatic carcinogenesis.^25^ Wu et al^26^ used deep RNA sequencing of three paired HCC tumor and adjacent tissues to characterize AS events. They identified only three AS candidates with potential prognostic and therapeutic value in HCC. A coiled‐coil domain containing 50 splice variants was regulated by the HBx/SRSF3/14‐3‐3β complex, which promoted oncogenic progression of HCC via the Ras/FOXO4 signaling pathway,^27^ which represented a promising therapeutic target for HCC. In this study, we comprehensively evaluated HCC‐specific AS events, and showed that hundreds of ASs were HCC‐specific.

In our study, many splicing variants showed potential prognostic significance for patients with HCC. The abnormal proteins expressed by these AS events were strongly associated with HCC, and they may play a crucial regulatory role in HCC progression. Furthermore, these proteins may be potential therapeutic targets. To evaluate further the significance of these AS events, multivariate Cox analysis was used to determine eight PIs based on associations between OS and ASs. Receiver operating characteristic analysis resulted in an AUC for PI‐ALL of 0.993 for prediction of cancer status in patients with HCC. Furthermore, PI‐ALL was significantly associated with OS (*P* = 2.2e−16).

Splicing factors execute the splicing process. Therefore, alterations in splicing factor expression can directly result in aberrant splicing events. A previous study showed that there were significant differences in the expression of splicing factors between cancer tissue and normal tissue.^28^ Splicing factors have the potential to become oncogenes or pseudo‐oncogenes, which can promote carcinogenesis.^29^, ^30^ In our study, we identified 12 splicing factors that were abnormally expressed in HCC. Previous studies identified splicing factors associated with HCC, such as PFS,^31^ FMRP,^32^ YB‐1,^33^, ^34^ hnRNPH1,^35^ and SRp20.^36^, ^37^ However, these studies did not perform functional analyses.

In our study, we evaluated survival and developed a correlation network of splicing factor expression and ASs, which allowed for evaluation of the underlying mechanisms of splicing pathways in patient survival. Furthermore, the splicing networks associated with HCC showed distinct interactions between splicing factors and ASs. Notably, upregulation of survival‐related splicing factors in HCC was associated with poor OS. Thus, our findings contributed to general understanding of splicing patterns and their mechanistic correlation to splicing factors in HCC, which may assist in characterization of the underlying mechanisms of AS in HCC tumorigenesis. Functional analysis in our study implicated several pathways in which these alternatively spliced genes were enriched, which likely contributed to carcinogenesis and progression of HCC.

Our study suffered from some limitations. Similar to other studies, a lack of existing therapeutic strategies could cause biases. Furthermore, studies that include a larger population of patients with HCC would be valuable. In addition, although our study identified multiple splicing events that theoretically impacted HCC, further external validation of prognostic Cox model and functional consequences of these events is necessary.

In this study, we developed comprehensive profiles of splicing events in patients with HCC, and identified several survival‐associated splicing events that could be utilized for development of prognostic signatures. Furthermore, a potential regulatory network was established for the associations between splicing events and splicing factors. The results of this study may contribute to understanding of the function of RNA AS in HCC carcinogenesis.

## CONFLICT OF INTEREST

The authors report no conflicts of interest.

## AUTHOR CONTRIBUTIONS

All authors contributed to data analysis, drafting or revising the article, gave final approval of the manuscript, and agree to be accountable for all aspects of the work. The first two authors contributed equally to this work.

## Data Availability

The data that support the findings of this study are available on request from the corresponding author.
