# Supplementary figures and images for: Profiles of prognostic alternative splicing signature in hepatocellular carcinoma
Source: Cancer Med. 2020 Jan 24;9(6):2171–80. doi: 10.1002/cam4.2875 (PMC7064038; doi:10.1002/cam4.2875)

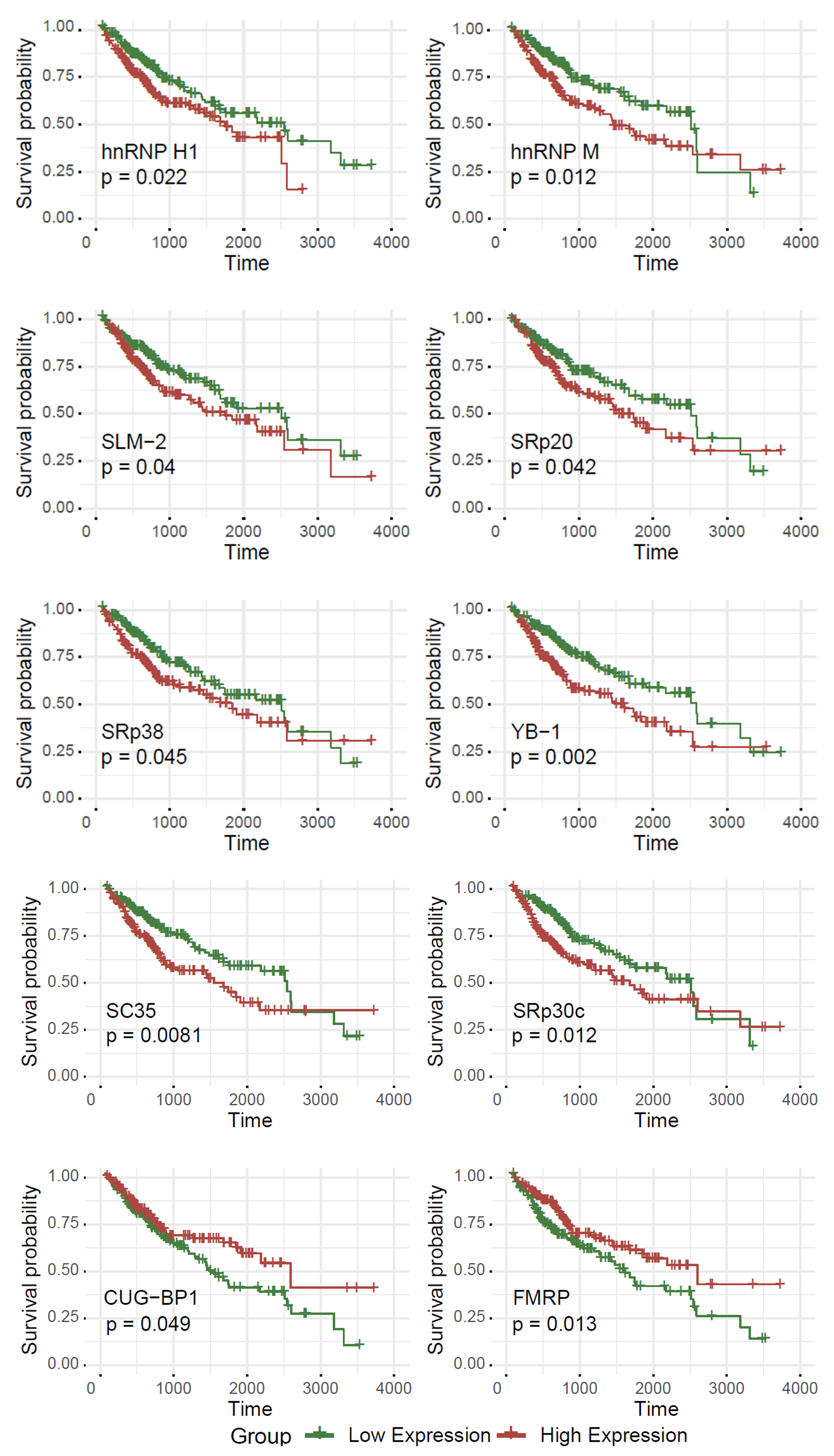

Supplement: Supplementary file 1 [file CAM4-9-2171-s001.tif]
